# Prevalence, Symptom Profiles, and Correlates of Mixed Anxiety–Depression in Male and Female Autistic Youth

**DOI:** 10.3390/neurosci5030025

**Published:** 2024-09-02

**Authors:** Vicki Bitsika, Christopher F. Sharpley, Kirstan A. Vessey, Ian D. Evans

**Affiliations:** Brain-Behaviour Research Group, University of New England, Armidale, NSW 2351, Australia; vicki.bitsika@une.edu.au (V.B.); kvessey@une.edu.au (K.A.V.); ievans3@une.edu.au (I.D.E.)

**Keywords:** autism, anxiety, depression, sex differences

## Abstract

Relatively little attention has been given to mixed anxiety and depression in autistic youth, particularly how this differs between males and females. This study investigated sex-based differences in the prevalence and correlates of mixed anxiety and depression in a sample of 51 autistic males (*M* age = 10.16 yr, SD = 2.81 yr, and range = 6 yr to 17 yr) and 51 autistic females (M age = − 10.07 yr, SD = 2.76 yr, and range = 6 yr to 17 yr), matched for age, IQ, and autism severity. Self-reports on generalised anxiety disorder and major depressive disorder, morning salivary cortisol, ADOS-2 scores, and WASI-II full-scale scores were collected from these autistic youth, and data on the ASD-related symptoms of these youth were collected from their parents. The data were analysed for total anxiety–depression score levels, for the underlying components of this scale, and for the individual items used in the scale. The results indicate no significant sex differences for the prevalence of mixed anxiety and depression total scores or the underlying components of anxiety and depression or for the individual items of the mixed anxiety–depression scale. There were sex differences in the significant correlates of mixed anxiety and depression: morning cortisol and ASD-related difficulties in social interaction for females, and ASD-related behaviour for males. Males’ feelings of being restless or edgy were correlated with their social interaction and repetitive and restricted behaviour. Females’ difficulties in social interaction were correlated with their concerns about their abilities and their sleeping problems. Females’ sleeping problems, their tendency to talk about dying, and feeling worthless, were correlated with their morning cortisol. These findings suggest that, while mixed anxiety and depression is experienced similarly by autistic males and females at the global, component, and individual item levels, specific aspects of the symptomatology of mixed anxiety and depression are differently associated with aspects of their ASD-related symptomatology and their levels of chronic physiological stress for males and females.

## 1. Introduction

### 1.1. Autism Symptomatology and Comorbidities

Autism spectrum disorder (ASD) is characterised by persistent deficits in social communication and social interaction plus restricted, repetitive patterns of behaviour [1]. Children and adolescents with ASD (hereafter, “autistic youth”) may also have related neurological, cognitive, psychiatric, and physical conditions [2]. In particular, neurodevelopmental disorders such as ADHD, language disorders, and intellectual disability [3] are also comorbid with ASD and can impede normal development. As well as these disorders that are neurologically related to ASD, many autistic youths also suffer from mental health disorders such as anxiety and depression. Anxiety disorders are found in approximately 40% of autistic youth [4], although some reports suggest that this figure might be as high as 82% [5]. Similarly, the prevalence of depression in autistic youth has been reported to be as high as 83.3% [6].

### 1.2. Measuring Anxiety and Depression in Autism

These data highlight the importance of the presence of anxiety and depression when assessing autism in youth, but there is some uncertainty about how best to achieve this. Central to this issue is the common practice of considering anxiety and depression as separate disorders, whereas they may be considered as points on a continuum of social distress [7,8,9]. For example, they share some symptoms, common psychophysiological pathways, and causal linkages to worry, prolonged arousal, lowered immune functioning, fatigue, low-level infections, feelings of helplessness, and pessimism that the future will offer any respite [1]. These commonalities have led to the consideration of a “mixed anxiety and depression” diagnosis [10], with specific treatment implications [11]. Although there has been some redefinition of mixed anxiety and depression into a form that is either primarily anxiety plus some depression symptoms, or primarily depression plus some anxiety symptoms [1], these do not include the whole range of symptoms for anxiety or depression, and thus may inadvertently miss some individuals who have particular sets of symptoms. Instead, measuring the presence of the range of symptoms that comprise the DSM-5-TR [1] diagnostic criteria for generalised anxiety disorder (GAD) and major depressive disorder (MDD) could provide a much more encompassing measure of the prevalence, and structure, of anxiety and depression.

### 1.3. Sex Differences

Although it has been traditionally estimated that there are more males than females who are autistic [12,13], there have been some recent comments that this may be due to autistic females camouflaging their autistic symptomatology [14], or the exclusion of females from research into ASD [15], rather than an actual disparity between males and females. Consequently, there has been some focus on comparisons between male and female autistic symptoms [16,17]. Because anxiety and depression are so often comorbid with ASD [4,6], it is of direct relevance in understanding the mental health of autistic youth and adolescents to compare the incidence of mixed anxiety and depression across male and female autistic youth and adolescents.

Most studies of anxiety and depression in autistic youth have recruited males only [18]. In the few studies (to May 2024) that have included samples of young autistic males and females, results have consistently indicated no significant sex differences in anxiety or depression [4,19,20]. However, there are some methodological limitations in those studies that limit the generalisability of the reported results. For example, Ambrose et al. [4] matched their male and female participants on age, receptive language, and some aspects of the ASD diagnostic criteria, but they were not matched on their IQ, despite IQ being previously associated with anxiety in autistic youth [21], although with some degree of inconsistency [22]. Similarly, age has been significantly associated with anxiety in autistic youth, with younger individuals (6–11 years) exhibiting higher levels of GAD than older youth (12–18 years) [23], suggestive of a need to compare these two age subgroups for their anxiety severity. A recent study of sex differences in depression scores of autistic and non-autistic adolescents did not include younger youth in their sample [20], and a review of the prevalence of a range of psychiatric symptoms in autistic youth did not find sufficient reports of males and females to include sex as a variable [24]. One study did match autistic males and females on age and IQ [19] and found no significant sex differences in the total anxiety and depression inventory scores, but the scales used were not completely based on the GAD and MDD diagnostic criteria and had some psychometric problems [25], and no data were reported on individual symptom profiles. Finally, all of these studies examined either or both anxiety and depression, but not mixed anxiety and depression.

### 1.4. Correlates of Mixed Anxiety and Depression

Although identification of the profiles of mixed anxiety and depression across autistic male and female youth is valuable in itself for further clarification of the relative symptomatology of the two sexes in this population, there is also some potential for focused clinical treatments by testing for possible associations between mixed anxiety and depression and other variables that may be linked with the GAD and MDD prevalence in autistic youth. For example, GAD and/or MDD have been correlated with ASD symptom profiles [18,26,27], but this has not been carried out for mixed anxiety and depression in autistic males and females.

Similarly, GAD and MDD are often found to emerge from periods of prolonged stress in autistic samples [28], which reflects autonomic dysfunction that is evident in the prolonged activation of the hypothalamus–pituitary–adrenal (HPA) axis, the end product of which is cortisol [29,30]. In particular, elevated levels of salivary cortisol have been found to correlate with anxiety [31] and depression [32].

### 1.5. Aims of This Study

Therefore, because the combination of anxiety and depression can provide a more comprehensive diagnostic evaluation of these problems in autistic youth, and because individual GAD and MDD symptom profiles are more informative in developing personalised treatment plans than total inventory scores, this study aimed to test for sex differences in mixed anxiety and depression at the total score, component scores, and individual symptom levels in autistic males and females aged between 6 years and 18 years and matched for age and IQ. On the basis of previous research, the null hypothesis, that there would be no significant sex differences for any of these three anxiety–depression metrics, was tested. Because previous research has shown that anxiety and depression are correlated with various aspects of ASD symptomatology, and also HPA axis stress indicators (i.e., cortisol), it was hypothesised that mixed anxiety and depression would be correlated with the autistic participants’ ASD-related symptomatology and also their salivary cortisol levels.

## 2. Materials and Methods

### 2.1. Participants

Sufficient participants were drawn from a larger study about anxiety and depression in autistic males and females [33] to provide a sample of 51 autistic females (*M* age = 10.07 yr, SD = 2.76 yr, and range = 6 yr to 17 yr) and 51 autistic males (*M* age = 10.16 yr, SD = 2.81 yr, and range = 6 yr to 17 yr) and 1 of each of their parents. These participants were recruited from parent support groups and other support organisations on the Gold Coast, Australia, via advertising and personal presentations by the first author to those groups. All these males and females had previously been diagnosed as autistic by a registered paediatrician or psychiatrist, confirmed by administration of the ADOS-2 [34] by a research-competent staff member during recruitment to this study (all the young autistic males and females had ADOS-2 scores of >7). There were no significant differences in either age (*F*(1,101) = 0.020; *p* = 0.887) or IQ (males: *M* = 97.9, SD = 12.0, and range = 76–125; females: *M* = 98.2, SD = 13.1, and range = 77–128: *F*(1,101) = 0.006; *p* = 0.937) between these males and females.

All these autistic participants were attending mainstream schools and were capable of self-care appropriate to their age. The females’ parents reported that 14 of their daughters had reached menarche, but biological tests for puberty were declined by the females and the males because they considered them to be too intrusive. There were no significant differences for the mixed anxiety and depression scores between females who had reached menarche and those who had not (all *p* > 0.09), and so all the females’ data were analysed together.

### 2.2. Age

Because of the lack of biological data verification of puberty for both males and females and because guessing the presumed age of puberty may be inaccurate due to variable maturation rates, it was decided to test for age effects by correlational procedures. Additionally, age is a continuous variable, and although it would be valuable to divide the samples into child versus adolescent, dichotomisation can have limiting effects on the reliability and power of statistical procedures [35,36].

### 2.3. Instruments

#### 2.3.1. Autism

The Autism Diagnostic Observation Schedule Second Edition (ADOS-2) [34] was used to confirm a diagnosis of ASD via an overall total SA + RRB score of 7 or more, as recommended by the ADOS-2 authors [34].

The ADOS-2 is recommended for the purpose of classifying ASD severity [37,38] and has demonstrated a sensitivity of 0.89 to 0.92, a specificity between 0.81 and 0.85 [39], and a test–retest reliability of 0.71 to 0.89 [40].

#### 2.3.2. IQ

The Wechsler Abbreviated Scale of Intelligence (2nd Edition) (WASI-II) [41] is a screening test of intelligence with established validity with the WISC-IV when used with autistic participants [42]. The WASI-II consists of four subtests (vocabulary, similarities, block design, and matrix reasoning) with reliability coefficients of between 0.92 and 0.96, and these subtests are used to calculate the full-scale IQ score. The recommendations of the authors of the WASI-II to use T-scores rather than raw scores from the four subtests to avoid any confounds due to age were followed ([41], p. 32).

#### 2.3.3. Anxiety and Depression

The fourth edition of the Child and Adolescent Symptom Inventory (CASI-4) [43] was used to assess generalised anxiety disorder and major depressive disorder as they are defined in the DSM-5-TR [1], and these were combined to provide a score for mixed anxiety and depression. The autistic males and females self-rated their symptoms according to frequency (0 = never, 1 = sometimes, 2 = often, and 3 = very often) as required by the CASI-4 authors. The CASI-4 has been used with autistic youth [44], and the CASI-4 test manual provides normative data for this population [43], and the CASI-4 is rated as one of only four scales that are appropriate to measure anxiety in autistic youth [45]. The CASI-4 GAD and MDD subscales have a reported internal consistency of 0.74 each [43].

The mixed anxiety and depression metric will be referred to from hereon as GAD-MDD. The possible range of scores on the GAD-MDD was between 0 and 48 because there were 16 discrete CASI-4 items included in the GAD-MDD scale. Two items were common to both CASI-4 subscales (I feel irritable most of the day; I don’t have much energy or I feel tired for no reason), but these were included only once each in the GAD-MDD scale because of the focus upon a combined anxiety–depression metric rather than two separate measures.

#### 2.3.4. Source of GAD-MDD Data

It has been demonstrated that parents’ own GAD or MDD can bias their assessment of their autistic child on these disorders [46,47]. Autistic youth of the ages recruited in this study can provide self-evaluations of their own anxiety that are more strongly correlated with a neurobiological index of stress and anxiety than their parents’ evaluations of their child’s anxiety [48]. Other data also support the use of self-report of depression by autistic youth and adolescents [49,50]. Therefore, self-assessment of the autistic males’ and females’ GAD-MDD was used in this study. Where needed, parents read the GAD-MDD items to their children but did not participate in the responses given by their children.

#### 2.3.5. The Autism Spectrum Disorder Behaviour Checklist (ASDBC)

The ASDBC [51] identifies the presence of ASD-linked symptoms exhibited by their child by asking parents to complete 30 items on a “present” (score of 1) vs. “absent” (0) basis. The ASDBC’s items include sections on communication, social interaction, and adaptive behaviour [1], and the total scores from each of these three sections were used in this study. Criterion validity has been established for the ASDBC with the Childhood Autism Rating Scale [52] (*r* = 0.71; *p* < 0.01) and the Adaptive Behaviour Composite scores from the Vineland Adaptive Behavior Scale [53] (*r* = 0.60; *p* < 0.01). The internal consistency is satisfactory for research purposes, as indicated by a Cronbach’s Alpha of 0.77 [54].

### 2.4. Saliva Assays

By the use of Salivettes (Sarstedt Australia, Mawson Lakes, Australia), approximately 1 mL of saliva was collected from each of the autistic males and females and assayed by a specific salivary cortisol ELISA kit from Abnova Corporation (KA1885, Taipei, Taiwan), which is a solid-phase ELISA using a polyclonal rabbit antibody directed against cortisol. Endogenous cortisol in the sample competes with a cortisol–horseradish peroxidase conjugate for binding to the antibody. This ELISA has an intra-assay variability of 8.27% and inter-assay variability of 8.33%, with a spiking recovery of 100% and a calibration range of 0.1 to 30 ng/mL. All standards, controls, and samples were assayed in duplicate, and the results were calculated using a 4-parameter logistics curve fit.

### 2.5. Procedure

Data were collected in the autistic participants’ homes to reduce the likelihood of extraneous anxiety. These participants were given verbal and written instructions for the completion of the GAD-MDD subscales (the autistic youth), the background questionnaire on age (and menstrual status for the autistic females) and sex at birth, and the ASDBC (for parents). In addition, saliva (from the autistic youth) was collected about 45 min after the autistic youth awoke in the morning (called “morning cortisol”). This process was modelled for all participants, and the participants were visited the day after the data collection to ensure that the procedure had been followed. The Salivettes were frozen in the participants’ homes until collected and then transferred to a −80 °C freezer until assayed by the ELISA protocol. ADOS-2 and WASI-II assessments were conducted within two weeks before these procedures. All parents gave written informed consent to participate, and their children gave written or verbal consent, depending on their ages. Ethical approval for these procedures was received from the Bond University Human Research Ethics Committee (BUHREC) in accordance with the Helsinki Declaration of 1964 (approval no. RO1516).

### 2.6. Statistical Analysis

The data were analysed via IBM SPSS 27 and JASP. The mean, SD, 5% trimmed mean, ranges, skewness, and kurtosis values, plus the internal consistency, were obtained for the GAD-MDD data. The normality of these scales was assessed by the Kolmogorov–Smirnov statistic. As noted by Stevens ([55], p. 6) when the sample size is 100 or more, “power is not an issue”, and so the current sample size was accepted as satisfactory for the purposes of this study. The effects of age and IQ on GAD-MDD were assessed by Pearson correlations for males and females, separately. Because of the large number of correlation coefficients this would produce, potentially risking a type I error, plus the equally risky procedure of applying a blanket correction (e.g., Bonferroni) that may have inflated the risk of a type II error [56,57], the yardstick for a "meaningful" correlation was set at an *r* of at least 0.3 (i.e., a medium-strength association) plus *p* < 0.05. ANOVA tested for differences in the GAD-MDD total scores for the females who had reached menarche (*n* = 14) and those who had not (*n* = 39) and for females versus males. Testing for sex differences in GAD-MDD was undertaken by Bayesian statistics using the JASP software version 0.19 [58]. Principal component analysis was used to identify the underlying components of the GAD-MDD data.

## 3. Results

### 3.1. Data

The internal consistency (Cronbach’s alpha) for GAD-MDD for all items was 0.895. The 5% trimmed mean for the GAD-MDD total scores for the males was 14.21, and for the females, it was 16.04, indicative of minimal effects from outliers (see Section 3.2.1 below). The skewness and kurtosis were within acceptable limits, confirmed by the histograms, normal Q–Q plots, and detrended Q–Q plots. The Kolmogorov–Smirnov and Shapiro–Wilk statistics were not significant for either males or females, allowing statistical analysis by Pearson’s product-moment correlation, ANOVA, and MANOVA.

The mean scores for the three ASDBC subscales, plus morning cortisol values, are shown in Table 1, indicating no significant sex-based differences for the ASDBC, and confirming the similarity of the males and females in terms of their ASD symptomatology severity. The females had significantly higher morning cortisol concentrations than the males.

### 3.2. Sex Differences in GAD-MDD

Sex differences in GAD-MDD were examined at three levels of increasing detail: total GAD-MDD scores; the underlying components of GAD-MDD; and individual GAD-MDD items. There were no meaningful correlations between GAD-MDD total scores and age, IQ, ADOS-2 scores, or menarche for autistic females, and there were none between GAD-MDD and age, IQ, and ADOS-2 scores for autistic males (all *p* > 0.08).

#### 3.2.1. Total GAD-MDD Scores

The mean total score of the males’ self-rating on GAD-MDD was 14.35 (SD = 6.38; range = 3–30); for the females, it was 16.24 (SD = 8.64; range = 1–38). There was no significant difference between these scores (t(100) = 1.251; *p* = 0.214; Cohen’s d = 0.248; BF _10_ = 0.419).

#### 3.2.2. Factor Structure of GAD-MDD

The total GAD-MDD scores provided an overall measure of this construct with these samples of autistic youth, and the individual GAD-MDD items enabled an analysis of these sex differences at the most detailed level. However, when scales are composed of a number of different items, it is of interest to examine those scales at the underlying component level, or how items group together to form a representation of a "factor" within the scale. The identification of sex-based differences, and correlates, of these factors can provide further insights into the profiles and correlates of GAD-MDD with autistic youth, and so this process was performed here via exploratory factor analysis because there are no previous reports of the factor structure of GAD-MDD in autistic youth of both sexes [59] and because it has been suggested that this method produces the most accurate parameter estimates [60]. The total sample size of 102 was used, as this is considered to be satisfactory for exploratory factor analysis [61], but the more important issues were those relating to the presence of item–item correlations of at least 0.3, and the presence of a Kaiser test value of 525.917, *p* < 0.001, and many item–item correlations of at least 0.3.

The principal component analysis indicated a three-factor solution, confirmed by the scree plot and parallel analysis. This three-factor solution explained 52.09% of the variance (factor 1 = 32.36%, factor 2 = 11.67%, and factor 3 = 8.06%). These three factors were only weakly correlated (all r < 0.288) and so were considered orthogonal. After varimax rotation, the three factors were seen to load discretely onto only a single GAD-MDD item each, thus providing a simple structure [62]. Table 2 shows the factor structure of the GAD-MDD scale for the 51 autistic males and 51 autistic females. The first factor was identified as anxiety and depression, factor 2 was named somatic and cognitive, and factor 3 was called fatigue/anhedonia. There were no significant sex differences for any of these three GAD-MDD factors (factor 1: t(100) = 1.040, *p* = 0.301, Cohen’s d = 0.206, and BF_10_ = 0.338; factor 2: t(100) = 1.597, *p* = 0.114, Cohen’s d = 0.316, and BF_10_ = 0.646; factor 3: t(100) = 0.000, *p* = 1.000, Cohen’s d = 0.000, and BF_10_ = 0.209). That is, whilst independent-sample *t*-tests indicated no gender effect for any factor (*p* = 0.301/0.114/1.000, respectively), Bayesian independent-sample *t*-tests were also performed to estimate the likelihood of gender effects for each of the three main factors via JASP (JASP Team, 2024). Assuming the prior was a Cauchy distribution centred at 0 with a width of 0.65 (corresponding to an 80% probability of an effect size between −2 and 2), anecdotal–moderate evidence for no gender effect (i.e., evidence for H_0_) was found (BF_10_ = 0.361 for factor 1, 0.683 for factor 2, and 0.225 for factor 3).

#### 3.2.3. Individual Item Comparisons

**Figure 1:**
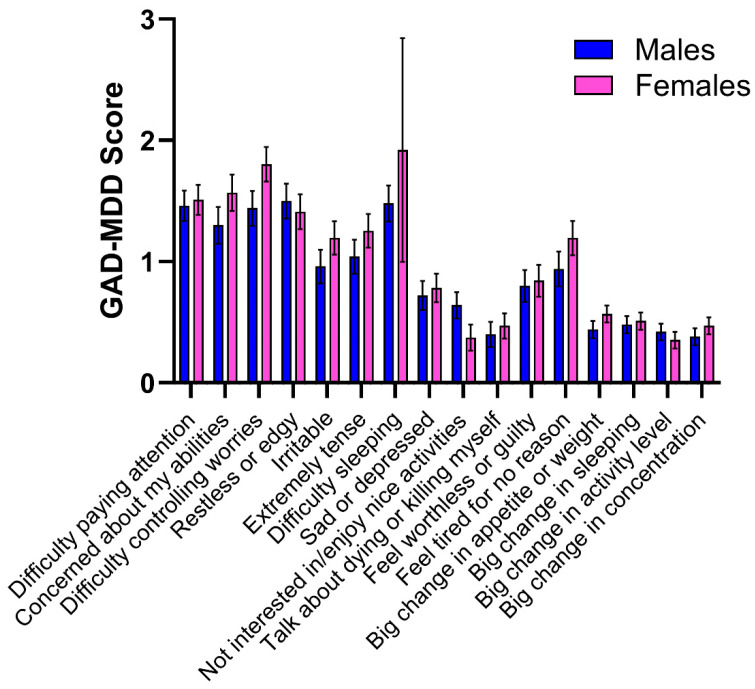
Mean (SE) of each of the GAD-MDD items for 51 autistic males and 51 autistic females.

Figure 1 shows the non-significantly different mean and standard error scores for males and females on all 16 items for GAD-MDD. The independent-sample *t*-tests ranged from t(100) = 0.105, *p* = 0.916, Cohen’s d = 0.021, and BF10 = 0.210, to t = 2.263, *p* = 0.026, Cohen’s d = 0.448, and BF10 = 1.981. The latter item (I have difficulty falling asleep or staying asleep at night) showed evidence of an anecdotal effect, as per the Bayes Factor Robustness Check (BF10), but not to a sufficient degree to warrant exclusion from the overall results.

### 3.3. Correlates of GAD-MDD

The correlation matrices for the autistic males and females indicated some sex-based differences in the ways that each of the two sexes’ GAD-MDD was related to various aspects of their ASD symptomatology and salivary cortisol. At the total GAD-MDD score level, the only meaningful correlation for females was between total GAD-MDD scores and morning cortisol (r = 0.395; *p* = 0.004), whereas for autistic males, it was between their total GAD-MDD score and their ASDBC behaviour score (r = 0.337; *p* = 0.017).

None of the three GAD-MDD factors were meaningfully correlated with any of the three ASDBC subscale scores, ADOS-2 score, IQ, or morning cortisol for autistic males. For females, morning cortisol was meaningfully correlated with the anxiety and depression factor (r = 0.360; *p* = 0.009) and also with the fatigue/anhedonia factor (r = 0.344; *p* = 0.013).

When considered at the individual GAD-MDD item level, males had meaningful correlations between restless or edgy and their ASDBC social interaction (r = 0.329; *p* = 0.018) and ASDBC behaviour (r = 0.328; *p* = 0.019) subscales. Females had a meaningful correlation between their GAD-MDD item concerned about my abilities and their ASDBC social interaction subscale; between difficulty sleeping and morning cortisol (r = 0.344; *p* = 0.014) and their ASDBC social interaction subscale (r = 0.350, *p* = 0.012); between talk about dying or killing myself and morning cortisol (r = 0.473; *p* < 0.001; and between feel worthless or guilty and morning cortisol (r = 0.388; *p* = 0.005).

## 4. Discussion

### 4.1. Hypothesis 1: Sex Differences

The first (null) hypothesis tested in this study was confirmed by the finding of no meaningful sex differences at the total score level, underlying the component level, or for the individual GAD-MDD items. Thus, these findings are congruent with the previous literature in finding no significant differences between male and female total anxiety and depression, but this study extends that previous finding in two ways. First, by examining the mixed anxiety and depression construct instead of GAD and MDD separately, and second, by including greater detail regarding this construct in the form of underlying components and individual symptoms.

The value of using mixed anxiety and depression rather than GAD or MDD separately lies in the overlap in the symptomatology of GAD and MDD. As well as sharing some symptoms, GAD and MDD are amenable to treatment with some of the same medications, share the same neurotransmitters, and are both more likely to occur following chronic stress [63]. However, despite this degree of overlap, they are not completely identical. Thus, including them both in the mixed anxiety and depression construct allows the non-overlapping aspects of GAD and MDD to be assessed and diagnosed, if present. On the basis of this (and previous) research, it is reasonable to expect no meaningful differences in the prevalence of mixed anxiety and depression between autistic male and female youth when considered at the total scale level, the underlying component level, or at the individual GAD-MDD item level. The single item regarding sleeping did not show more than an anecdotal difference, which did not justify it being identified as exhibiting significant sex differences.

Although the aims of this study did not include the comparison of autistic versus non-autistic young males and females, it is relevant to note that there was an odds ratio of 1.95 for MDD being more prevalent in young females across all ages and nations, appearing at the age of 12 yr [64]. There is some evidence that GAD is more prevalent among non-autistic boys at the age of 6 yr, but GAD is nearly three times more prevalent in girls by the age of 14 yr [65]. The comparative lack of sex differences for GAD or MDD here, plus the established elevated severity of GAD and MDD in autistic versus non-autistic youth [66], further argues for the consideration of anxiety and depression in young autistic people as being a unique and clinically relevant phenomenon.

Finally, whilst gendered effects can never be entirely ruled out, it does not appear to be a noteworthy factor in these results. The Bayes Factor Robustness check (JASP Team, 2024) indicated that the only evidence for a gendered effect would require a Cauchy distribution width of less than 0.27, and that would only apply to a single factor (factor 2). All other assumptions pointed towards stronger evidence for no gender effect; however, it should be acknowledged that Cauchy prior widths between 0.4 (factor 3) and 1.6 (factor 2) would be required to reach at least moderate evidence in favour of the null hypothesis (i.e., BF_10_ scores below 1/3).

### 4.2. Hypothesis 2: Correlates of GAD-MDD

The most interesting sex differences found in this study were in the testing of the second hypothesis, i.e., that ASD symptomatology and cortisol would be significantly correlated with GAD-MDD. The finding that, at the total GAD-MDD score level, females’ severity was meaningfully associated with their elevated cortisol levels suggests that this neurophysiological factor may have been implicated in the development of their anxiety–depression (although no directional causal process can be posited from these data). Chronic stress is established as a predecessor of GAD [28] and MDD [30], and this was confirmed for the females examined here. However, it was not meaningfully correlated with the total GAD-MDD score for males, who, instead, exhibited a meaningful correlation with one of the core diagnostic criteria for ASD-restricted and repetitive behaviour. These findings express a sex-based difference in GAD-MDD among autistic youth not previously reported and suggest different pathways to anxiety and depression between autistic male and female youth, although that kind of chronological causal progression cannot be assumed from this study.

When examined at the GAD-MDD component level, females, again, demonstrated the influence of chronic stress in the form of salivary cortisol. The meaningful positive correlations between morning cortisol and the anxiety and depression factor, and also the fatigue/anhedonia factor, confirm and extend the findings for the total GAD-MDD score for females by excluding one of the three components (somatic and cognitive), but the examination of the associations at the individual GAD-MDD item level provides the most valuable information.

Again, males’ GAD-MDD was linked with their ASD-related symptomatology (social interaction, and restricted and repetitive behaviour) but only for the symptom of feeling restless or edgy, whereas the females’ major correlate was morning cortisol (for three GAD-MDD items: difficulties sleeping, talking about dying or killing themselves, and feeling guilty or worthless). Although concern about their abilities and sleeping problems were linked with their ASD-related criteria difficulties in social interaction and repetitive and restricted behaviour, morning cortisol was also meaningfully associated with the symptoms of thinking about death and feeling worthless.

Overall, these correlational results imply that females’ GAD-MDD was linked with their HPA axis hyperactivation, most likely an outcome of chronic stress, whereas males’ GAD-MDD was connected with their ASD core diagnostic symptomatology. Previously unreported, these findings cast new light upon sex differences in anxiety and depression in autistic youth and further support the need to consider males and females as different in several basic ways in these often-comorbid disorders of anxiety and depression.

### 4.3. Limitations

Several limitations must be mentioned. First, the study sample was satisfactory for the statistical analyses, but greater power from increased sample sizes would add to the generalisability of these results. Second, the autistic males and females were volunteers, and no generalisation can be made to autistic youth who did not wish to participate in this study. Third, the sample was purposely restricted to autistic youth with an IQ of at least 70 and who were described as self-caring; other more severely disabled autistic youth may not report the same data as found in this study. Fourth, like all "snapshot" studies, no implications can be made for how these associations and prevalences might change over time, or during the normal development process, although there was no significant effect due to age on any of the variables studied here. Fifth, the self-reports of their GAD-MDD were used here because of the previous findings that these can be more valid than parental reports of their children’s anxiety or depression (Section 2.3.4), but it should be acknowledged that despite repeated findings that autistic children and adolescents can self-evaluate various internal states, such as loneliness [67], anxiety [68], and depression [69], caution should always be maintained when adopting this methodology.

Finally, the participants in this study declined physiological examination to establish puberty, so the effects of that factor upon the prevalence and correlates found here could not be investigated. Future research that accommodates these limitations will extend the generalisability of these findings. Several strengths of this study include the use of an established inventory for GAD and MDD; the use of self-reports of GAD and MDD rather than observers’ reports; the inclusion of a range of ASD-related and physiological correlates so that a wide spectrum of possible contributing variables was examined; and the investigation of the GAD-MDD construct at three levels of increasing detail.

## 5. Conclusions

Sex differences among autistic youth remain a key focus of research in their own right, as well as contributing to further understanding of the apparent discrepancy in the prevalence of ASD between males and females. Although the possibility that autistic females camouflage their ASD-related behaviour remains a major possible variable, it is also of benefit to consider how the often-comorbid disorders of GAD and MDD, and their mixed form, occur in autistic males and females. Information about the prevalence, structure, and correlates of GAD-MDD can help in understanding sex differences, as well as provide more factual bases for the diagnosis and treatment of ASD-related behaviour.

## Figures and Tables

**Table 1:** Mean (SD) scores for three ASDBC ^1^ subscale scores and morning salivary cortisol ^2^ for 51 autistic females and 51 autistic males.

	Males	Females	*F*	*p*
ASDB communication	13.92 (5.57)	13.90 (5.32)	0.000	0.987
ASDBC social interaction	17.46 (6.950	18.14 (5.98)	0.276	0.601
ASDBC adaptive behaviour	15.64 (5.90)	14.92 (5.16)	0.425	0.516
Morning cortisol	10.56 (6.26)	20.32 (13.16)	22.095	<0.001

^1^ Autism spectrum disorder checklist [51]; ^2^ nmol/L.

**Table 2:** Factor structure of GAD-MDD scale for 51 autistic females and 51 autistic males.

GAD-MDD Symptom	Factor 1	Factor 2	Factor 3
Feel worthless or guilty	0.828		
Difficulty paying attention	0.736		
Extremely tense	0.731		
Talk about dying or killing myself	0.71		
Difficulty controlling my worries	0.684		
Restless or edgy	0.684		
Irritable	0.682		
Concerned about my abilities	0.642		
Sad or depressed	0.631		
Big change in sleeping		0.793	
Big change in concentration		0.739	
Big change in appetite or weight		0.645	
Big change in activity level		0.562	
Difficulty sleeping		0.482	
Not interested/enjoy nice activities			0.841
Feel tired for no reason			0.703

## Data Availability

The raw data supporting the conclusions of this article will be made available by the authors upon request.
